# Early EEG-burst sharpness and 2-year disability in extremely preterm infants

**DOI:** 10.1038/s41390-023-02753-5

**Published:** 2023-07-27

**Authors:** Anne Mette Plomgaard, Nathan Stevenson, James A. Roberts, Tue Hvass Petersen, Sampsa Vanhatalo, Gorm Greisen, the SafeBoosC-II study group, Adelina Pellicer, Monica Fumagalli, Petra Lemmers, Gerhard Pichler, Eugene Dempsey, Olivier Claris, Simon Hyttel-Sorensen

**Affiliations:** 1 Department of Neonatology, Copenhagen University Hospital, Rigshospitalet, Blegdamsvej 9, 2100 Copenhagen, Denmark; 2 Brain Modelling Group, QIMR Berghofer Medical Research Institute, Herston, Brisbane, QLD 4006 Australia; 3 THP Consult, Odense, Denmark; 4 BABA Center, Departments of Clinical Neurophysiology and Physiology, Children’s Hospital, Helsinki University Hospital and University of Helsinki, Helsinki, Finland; 5 Department of Neonatology, La Paz University Hospital, Madrid, Spain; 6 NICU, Fondazione IRCCS Ca’ Granda Ospedale Maggiore Policlinico, Milan, Italy; 7 University Medical Center Utrecht, Wilhelmina Children’s Hospital, Utrecht, The Netherlands; 8 Department of Pediatrics, Medical University of Graz, Graz, Austria; 9 Department of Paediatrics and Child Health, University College Cork, Cork, Republic of Ireland; 10 Department of Neonatology, Hopital Femme Mere Enfants, Bron, France

## Abstract

**Background:**

Automated computational measures of EEG have the potential for large-scale application. We hypothesised that a predefined measure of early EEG-burst shape (increased burst sharpness) could predict neurodevelopmental impairment (NDI) and mental developmental index (MDI) at 2 years of age over-and-above that of brain ultrasound.

**Methods:**

We carried out a secondary analysis of data from extremely preterm infants collected for an RCT (SafeBoosC-II). Two hours of single-channel cross-brain EEG was used to analyse burst sharpness with an automated algorithm. The co-primary outcomes were moderate-or-severe NDI and MDI. Complete data were available from 58 infants. A predefined statistical analysis was adjusted for GA, sex and no, mild–moderate, and severe brain injury as detected by cranial ultrasound.

**Results:**

Nine infants had moderate-or-severe NDI and the mean MDI was 87 ± 17.3 SD. The typical burst sharpness was low (negative values) and varied relatively little (mean –0.81 ± 0.11 SD), but the odds ratio for NDI was increased by 3.8 (*p* = 0.008) and the MDI was reduced by –3.2 points (*p* = 0.14) per 0.1 burst sharpness units increase (+1 SD) in the adjusted analysis.

**Conclusion:**

This study confirms the association between EEG-burst measures in preterm infants and neurodevelopment in childhood. Importantly, this was by a priori defined analysis.

**Impact:**

- A fully automated, computational measure of EEG in the first week of life was predictive of neurodevelopmental impairment at 2 years of age.
- This confirms many previous studies using expert reading of EEG.
- Only single-channel EEG data were used, adding to the applicability.
- EEG was recorded by several different devices thus this measure appears to be robust to differences in electrodes, amplifiers and filters.
- The likelihood ratio of a positive EEG test, however, was only about 2, suggesting little immediate clinical value.

## Introduction

Reliable early prediction of neurodevelopment in extremely preterm infants may be clinically relevant, and also used to select infants for therapeutic trials as well as for advancing understanding about the mechanisms that underlie preterm brain injury. Cerebral injury as seen by cranial ultrasound (cUS) and visually evaluated (a)EEG features are associated,^1^ and in one early study, EEG significantly predicted death or neurodevelopmental impairment in infants with significant peri- or intraventricular haemorrhage.^2^ A systematic review identified 13 studies in preterm infants using EEG or amplitude-integrated EEG in the first week of life to predict neurodevelopmental outcomes.^3^ The pooled sensitivity and specificity were both 0.83. The challenge, however, is that visually assessed (a)EEG features are based on subjective evaluation and require substantial training, which precludes their use as a widely scalable method for clinical or research use. In contrast, quantitative computational measures of neonatal EEG signals have been developed to provide solutions that are fully objective, can be automated, and have been shown to correlate with outcomes.^4–7^

The SafeBoosC-II randomised clinical trial demonstrated that it is possible to reduce the burden of cerebral hypoxia or hyperoxia in extremely preterm infants by monitoring cerebral oxygenation during the first 3 days of life.^8^ A previous secondary analysis, across the two treatment groups, demonstrated that cerebral hypoxia was associated with reduced EEG activity measured as a lower burst rate.^9^

Here, we exploited the SafeBoosC-II cohort with its EEG data and neurodevelopmental follow-up^10^ to assess whether measures of EEG-burst shape predict neurodevelopment. The automated EEG-burst metrics we used were originally developed in the context of burst suppression in term infants,^11,12^ and later shown to carry diagnostic and prognostic information on the distinct phenomenon of bursts in preterm EEG.^5,13^ These metrics are sensitive to the sizes and durations of bursts in the EEG, and to their shape—in particular, their symmetry and sharpness. The hypothesis was that increased burst sharpness would be associated with neurodevelopmental impairment and/or low cognitive scores. The aims were, unlike previous studies,^3^ to specify the choice of EEG measures and outcome variables in advance and to assess the value of EEG over-and-above the predictive value of cUS.

## Patients and methods

The SafeBoosC-II trial (NCT01590316) was a multi-centre randomised trial with eight centres and the study was conducted between June 2012 and December 2013.^8^ The inclusion criteria for the trial was gestational age less than 28 completed weeks, and the ability to start cerebral oximetry monitoring within 3 h after birth and parental consent. The exclusion criterium was the lack of a decision to provide full life support.

### Electroencephalogram

#### EEG recordings

EEG recording was specified in a standard operating procedure. At the postnatal age of 64 h (±8 h) at least 120 min of good quality EEG with aEEG tracing was to be recorded.^14^

#### EEG analysis

The device-specific EEG data were converted to a common Matlab format. We used a single P3–P4 derivation for analysis (or C3–C4 as its substitute). EEG recordings were heterogeneous; seven different devices were used:^14^ Micromed EEG system (Mogliano, Veneta, Italy, *N* = 1), Nervus monitor (Cephalon, Norresundby, Denmark, *N* = 19), Olympic cerebral function monitor (Natus, Pleasanton, CA, *N* = 12), BRM2 and BRM3 (Natus, former Brainz monitor, *N* = 17), NicoletOne video-EEG system (Carefusion, Madison, WI, *N* = 4), and g.recorder (g-tec, Graz, Austria, *N* = 5). Thus, there was variation in EEG amplifier model/filter settings and electrode positioning. To overcome this, we post-processed EEG recordings using the ‘lowest common denominator-principle’: the data was filtered with a second-order Butterworth high-pass filter at 1 Hz, and high-frequency noise was removed using a notch filter at 50 Hz, and a second-order Butterworth low-pass filter at 24 Hz. Multiple 1-h epochs (average number of epochs was six) were evaluated per recording and the median feature value across all epochs was used as the final summary measure of each feature per recording. EEG epochs were automatically assessed for excessively high or low amplitude artefacts. If more than 25% of the EEG epoch (evaluated per second) was over 500 microvolts or 0 microvolts then it was excluded. Out of the 147 EEG recordings that were available from the 166 trial participants, 118 (80%) passed this quality control.

All EEG analysis was conducted in Matlab version 2018b, without knowledge of the medical history of the infant.

#### Measures of bursts

**Fig. 1:**
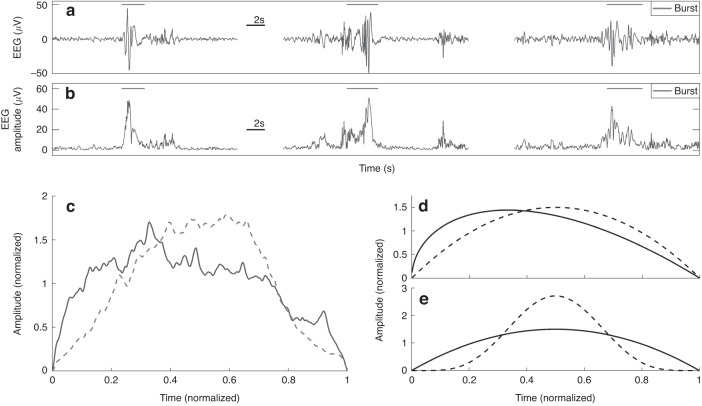
The principles of burst shape analysis. Single-channel EEG recording (**a**). Bursts of a select duration (2–4 s) were identified using the EEG amplitude signal (**b**) and averaged across 1 h of recording after normalising for amplitude and duration (**c**). The shape of the average burst is defined by its symmetry (**d**) and sharpness (**e**). Symmetry is calculated similarly to the skewness of a probability distribution, and sharpness is calculated similarly to the excess kurtosis of a probability distribution. The dashed lines in (**d**) and (**e**) represent increasingly symmetric and sharp burst shapes, respectively. The negative numbers for sharpness reported here are normalised to 0 by subtraction of 3 and this means that the mean burst shapes in this study were always less sharp, had less ‘peak-and-tails’ than the normal distribution. In (**c**), the average burst in an infant normal outcome (full line) and with neurodevelopmental impairment (dashed line) is shown.

The hallmark of preterm EEG is the spontaneously occurring activity transients (a.k.a. SAT), hereafter jointly called bursts^15^ and we chose features of burst-related metrics based on prior publications.^5,13^ First bursts were identified by the use of an amplitude threshold that maximises the number of bursts in each epoch of EEG. Next, bursts lasting 2–4 s were selected from the 1-h epoch of EEG and re-scaled to unit burst duration as well as unit average amplitude (burst area). All re-scaled bursts were then averaged to form a typical burst shape from which features of burst symmetry (skewness) and burst sharpness (kurtosis) were calculated (Fig. 1). Note that the negative numbers for sharpness reported here are normalised to 0 by subtraction of 3 and this means that the mean burst shapes in this study were always less sharp, had less ‘peak-and-tails’ than the normal distribution. We only used bursts of 2–4 s in duration as these have been shown to have diagnostic yield and correlation with age (c.f. Figures 3F and 5B in Iyer et al. 2015^13^ and Table S1 in Stevenson et al. 2020^4^). These metrics are dimensionless and thus invariant to the underlying EEG amplitude and frequency components of the bursts of the select durations.

Finally, a combinatorial feature was selected that uses measures of burst symmetry, burst sharpness, burst number and the slope of burst area vs. burst duration.^13^ The feature combination was defined as the first principal component from a kernel-PCA (Gaussian kernel with a gamma parameter = 1)^16^ and was derived from a previously studied, independent dataset.^4^

The three features were selected as they have all been shown to correlate with gestational age, presence of IVH, and measures of neurodevelopmental outcome.^4,5,13^ Out of them, at 72 h after birth, burst sharpness was best at predicting neurodevelopmental outcome in preterm infant,^13^ and therefore chosen as the ‘best candidate measure of burst shape, and it was expected that increased sharpness—in spite of the fact that sharpness typically has a negative value, i.e., has less ‘peak-and-tails’ that the normal distribution—would be associated with higher risk of poor neurodevelopmental outcome.

### Cranial ultrasound

In the SafeBoosC-trial cUS was also done at later stages, and at all ages, the scans were categorised in the same way as no, mild/moderate, or severe brain injury as previously described.^17^ No brain injury: None of the findings below. Mild/moderate brain injury: grade 1–2 IVH (including germinal layer haemorrhage) and isolated ventriculomegaly with ventricular index >p97.^18,19^ Severe brain injury: intraventricular haemorrhage grade III (ventricular index >p97 during the acute phase), post haemorrhagic ventricular dilatation, parenchymal/periventricular haemorrhagic infarction, unilateral porencephalic cysts, cystic periventricular leukomalacia (bilateral), cerebellar haemorrhage or stroke. But, for the present study, only data on cUS performed at day 1 and day 4 were considered and the latter pathologies are unlikely to have contributed significantly to the classification.

### Neurodevelopmental outcome

At 2 years corrected age, the participants were invited to a follow-up visit consisting of a medical examination and an assessment of their neurodevelopment. Medical examination: Basic growth measurements were collected. Vision and auditory functions were evaluated. If the child showed signs of cerebral palsy (CP), the gross motor function was classified using the Gross Motor Function Classification System (GMFCS). The doctor performing the medical examination may not have been blind to the intervention. Bayley Scales of Infant and Toddler Development, Second Edition (Bayley II) or Third Edition (Bayley III), depending on what version the centre was using at the time of the study. Bayley III is known to underestimate the developmental deficit when compared to Bayley II,^20^ we therefore calculated the predicted mental developmental index for the Bayley III edition, as previously described.^21^

Neurodevelopmental impairment was classified according to the recommendations of the British Association of Perinatal Medicine in 2008. Severe neurodevelopmental disability if any of the following conditions was present: CP with a GMFCS score of 3–5; a cognitive function score below –3 standard deviations from the mean, Mental Development Index (MDI <55); hearing impairment with no useful hearing even with aids; no meaningful words or signs; or blind or only able to perceive light or light reflecting objects. Moderate neurodevelopmental disability if any of the following conditions was present: CP with a GMFCS score of 1–2; a cognitive function score between –3 and –2 standard deviations from the mean (MDI 55–70); hearing impairment, but useful hearing with aids; fewer than five words or signs; or moderately impaired vision.

### Statistics

The statistical analysis plan for the secondary analysis of EEG at 64 h of age to predict neurodevelopmental outcome at 2 years corrected age in the SafeBoosC-II trial was completed before any data analysis.

For the reasons given above, EEG-burst sharpness was chosen as the best candidate for predictive ability. It was considered relevant to test a binary measure of neurodevelopmental outcome (the presence of moderate-or-severe disability) as well as a continuous measure (mental developmental index). Since there were two outcomes, a *p* value of less than 0.025 in any of them is used to claim the significant predictive ability of EEG. Supplementary analysis was to be unrestricted and exploratory and used to direct further research.

The aims of the prespecified statistics analysis plan were as follows:

1. To quantify ‘conventional’ ultrasound-based prediction of moderate-to-severe neurodevelopmental impairment at 4 days of age, a multiple logistic regression was conducted including gestational age above or below 26 weeks and sex as forced entry and brain injury diagnosed by cUS classified as none, mild–moderate, or severe within the first 4 days as independent variables. A step-down procedure was used to eliminate independent variables until a threshold of *p* < 0.1 was reached.
2. To investigate the added predictive value of EEG at 64 h over-and-above the final set of independent variables, a multiple logistic regression was done, by inclusion of the EEG-burst sharpness as an extra independent variable.
3. To investigate the added predictive value of EEG on cognitive development, a multiple linear regression was performed with the Bayley II mental developmental index equivalent as a dependent variable and the same independent variables as above. The same step-down procedure was used and EEG-burst sharpness was added as an extra independent variable.
4. A receiver operating curve was constructed using EEG-burst sharpness at different cut-offs to predict moderate-or-severe neurodevelopmental outcome, unadjusted for other risk factors.
5. Supplementary analysis was done as indicated by the data, including testing more parameters of the EEG and other combinations of independent variables.
6. Dosing of opioids induces temporary suppression of EEG in preterm infants.^22^ It was not included, however, in the prespecified model. Therefore, since data on opioids administered within 3 h before the start of the EEG recording were available, this was included as a final step.

## Results

**Fig. 2:**
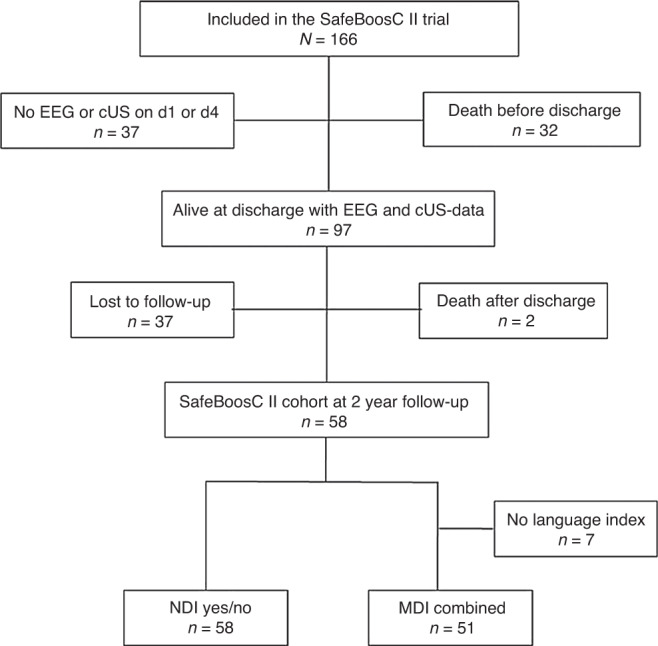
Study profile.

A total of 166 children were included in the SafeBoosC-II trial; of these, 97 had quality-wise successful EEG recordings as well as a cUS within the first 4 days of life and were alive at discharge. Of these children, only 58 participated in the follow-up programme at 2 years of age (Fig. 2). Mean gestational age was 26 w + 5 d ± 10 d SD and mean birth weight was 900 g ± 204 g SD. Of the 58 infants, 26 were males, 11 had gestational age below 26 weeks, 21 had moderate brain injury on cUS, only three had a severe injury on cUS, and 7 were exposed to opioids. As outcomes, nine of the 58 children had moderate-or-severe neurodevelopmental impairment, and the mean combined mental developmental index was mean 87.1 points ±17.3 SD.

EEG-burst sharpness in the 58 infants was –0.81 ± 0.11 SD, compared to –0.68 ± 0.18 in the 18 infants with EEG who died before discharge (*p* < 0.001), and to –0.81 ± 0.13 SD in the 37 infants who survived to discharge but were lost to follow-up (*p* = 1.0).

**Fig. 3:**
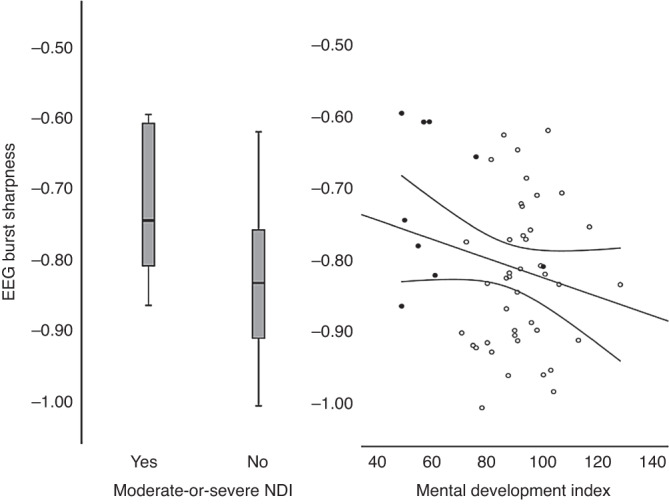
Neonatal EEG-burst sharpness in children born extremely preterm with and without neurodevelopmental impairment at 2 year of age (left) and as a function of mental development index (right—filled circles are children with neurodevelopmental impairment). Note: in the statistical analysis, the EEG-burst sharpness was the predicting variable and the analysis was adjusted for other predictors available shortly after birth.

**Fig. 4:**
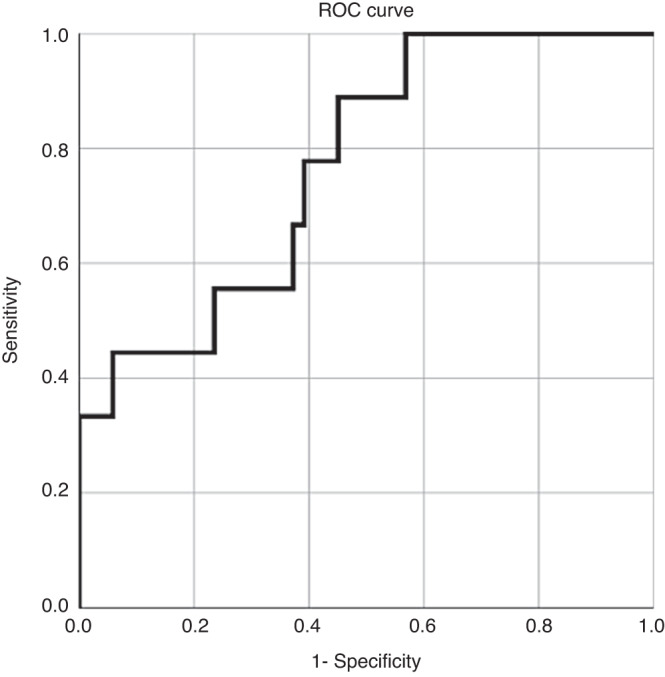
Receiver operating curve of the prediction of moderate-or-severe neurodevelopmental disability by the unadjusted EEG-burst sharpness. At a high threshold of burst sharpness, the sensitivity is 0.45, and the specificity is 0.85 and at a low threshold, the sensitivity is 0.9, but the specificity is only 0.5. Thus, a typical value of the likelihood ratio for a positive test is about 2.

As regards the prediction of NDI, of the conventional clinical risk factors, only mild–moderate brain injury was predictive (OR 4.5) (Table 1, model ‘Forced entry’ and model ‘Reduced’) perhaps since only three infants had severe brain injury. Adding EEG-burst sharpness of EEG at 64 h of age to the model improved the prediction of moderate-or-severe developmental impairment (OR 3.8 per 0.1 unit; *p* = 0.008; Table 1, model ‘+EEG’, Fig. 3). The robustness of the predictive value of burst sharpness was tested by varying the set of co-variates: First without any (*p* = 0.013), then with GA only (*p* = 0.016), and finally with GA and mild–moderate brain injury (*p* = 0.007). The area under the receiver operating characteristic (ROC) curve of the prediction of the binary outcome by the unadjusted EEG-burst sharpness was –0.77 (Fig. 4).

**Table 1:** Prediction of moderate-or-severe neurodevelopmental disability.

Predictor	Forced entry		Reduced		+EEG	
	OR	*p* value	OR	*p* value	OR	*p* value
Gestational age below 26 weeks	0.4	0.27				
Male sex	2.5	0.26				
Severe brain injury on cUS	0	0.99				
Mild or moderate brain injury on cUS	3.7	0.1	4.5	0.05	10.8	0.02
EEG-burst sharpness (per 0.1 unit)					3.8	0.008

The results of three pre-determined logistic regression models. EEG-burst sharpness has a predictive ability over-and-above the ‘conventional’ predictors (*p* = 0.008).

*OR* odds ratio, *cUS* cranial ultrasound.

GA below 26 weeks of gestation was significantly associated with MDI (Table 2, model 1 and 2). EEG-burst sharpness did not significantly improve the prediction (effect size –3.2 MDI points per 0.1 unit, *p* = 0.14, Table 2, model ‘+EEG’, Fig. 3).

**Table 2:** Prediction of mental developmental index.

Predictor	Forced entry		Reduced		+EEG	
	*b*	*p* value	*b*	*p* value	*b*	*p* value
Gestational age below 26 weeks	–15.1	0.017	–16.6	0.008	–16.3	0.008
Male sex	–1.8	0.70				
Severe brain injury on cUS	8.6	0.48				
Mild or moderate brain injury on cUS	–5.3	0.28				
EEG-burst sharpness (per 0.1 unit)					–3.2	0.14

The results of three pre-determined linear regression models. EEG-burst sharpness does not have predictive ability over-and-above the ‘conventional’ predictors.

*b* regression coefficient, *cUS* cranial ultrasound.

The administration of opioids up to 3 h before EEG recordings was associated with increased EEG-burst sharpness (–0.72 ± 0.10 SD vs –0.82 ± 0.10 SD, *p* = 0.02) but did not influence the prediction of neurodevelopmental impairment (odds ratio changed from 3.8 to 3.7, *p* = 0.31).

Neither EEG-burst symmetry (0.053 ± 0.10 SD vs 0.086 ± 0.11 SD, *p* = 0.4) nor the combinatorial EEG-burst feature (1.02 ± 1.33 SD vs 1.25 ± 1.21 SD, *p* = 0.25) was significantly associated with neurodevelopmental impairment, or the mental developmental index (*b* = 1.7 per 0.1 unit *p* = 0.5, and –0.09 per 0.1 unit, *p* = 0.7).

## Discussion

Our results show that an objectively and automatically measured property of preterm EEG-burst shape during the first days of life is predictive of childhood neurodevelopment, and this may go over-and-above that of other early perinatal variables, including cUS evidence of early brain injury.

Thirteen previous studies on the association between various measures of early EEG (first week) and neurodevelopmental outcome of preterm infants were included in a 2017 systemic review.^3^ Sensitivities ranged from 0.35 to 0.94 and specificities from 0.65 to 1.00. In our receiver operating curve sensitivity ranged from 0.45 to 0.90 and specificity from 0.5 to 0.85. So, our work on a computational measure of EEG is compatible with the results of previous studies, even under the strict regimen of an a priori approach.

Thus, the major strength of this study was that the EEG analysis was blinded to all clinical variables, the choice of primary and exploratory EEG features, the two co-primary neurodevelopmental outcomes and the statistical analysis used to test the associations were also all defined a priori. This adds to the credibility of the *p* values. Although the *p* value for the prediction of the cognitive score (0.14) was not statistically significant at the value of 0.025 chosen due to having two co-primary outcomes, the association was still in the expected direction as seen in Fig. 3 and the statistical significance as regards NDI was clear and robust in sensitivity analyses. Thus, we consider our overall result as statistically significant evidence in favour of our hypothesis. Furthermore, our study was a secondary analysis of data collected for a multinational randomised controlled trial, the devices used for recording of EEG varied and only single-channel EEG was used—this should all add to the generalisability. While only 80% of the EEG data passed quality controls, we expect that future prospective EEG studies could improve this rate. The loss of infants for analysis due to missing data was considerable, but the burst sharpness was higher in infants who died before discharge (our reason not to include death in a combined outcome in the first place was that predicting death in this group of infants in easier than predicting neurodevelopmental outcome). Furthermore, burst sharpness was very similar between those who were lost to follow-up and those included in the analysis so it is not obvious how this should have biased the results towards a false association.

The major weakness of the study is that the final sample size was only moderately sized. While the *p* value of the statistical association of the primary EEG feature (burst sharpness) to one of co-primary outcomes (moderate-or-severe neurodevelopmental impairment) was below 0.01, only nine infants had that outcome. This clearly limits the precision of any estimate of correlation as well as the reproducibility of the statistical adjustment for potential confounding factors and it would make subgroup analysis, e.g., of children with major NDI, meaningless. Nevertheless, our study still does provide independent evidence in favour of the hypothesis that increased burst sharpness in early EEG predicts poor neurodevelopmental outcome in preterm infants.^13^

Technically, EEG-burst sharpness has the virtue of being dimensionless, and due to the scaling of all bursts, it is not dependent on the EEG voltage scale nor burst duration (within the 2–4 s range that were included in the analysis), and hence is less sensitive to the filtering and frequency characteristics of the recording device than many traditional signal processing measures. The same goes for EEG-burst symmetry and the slope of the relationship between burst duration and burst area that were the components of the ‘combinatorial feature’. Moreover, the present kind of analysis can be fully automated and the burst detection individually adjusted,^13^ unlike the highly subjective and variable detection of bursts in the conventional visual review.^23^

Since the 2017 systematic review cited above, more studies have demonstrated associations between measures of early EEG power in preterm infants and cognitive scores in early^24^ and middle childhood.^25,26^ This may fit expectations, as EEG power is one measurable quantity of the magnitude of cortical activity, which is an important neural substrate for cognitive functions. Likewise, EEG-burst sharpness, as used in this study, may also characterise the local cortical activity. The weaker link of early burst shapes to later Bayley scores might sound disappointing to a clinician wishing to see specific bedside predictors. However, there is a very long leap with a myriad of mediating neuronal mechanisms between the early spontaneous cortical activity and the later neurocognitive performance. Finally, the combinatorial feature was developed by ‘training’ on a specific dataset, and the present negative finding may suggest overfitting to the training data.

This study was in extremely preterm infants. Prematurity is a continuum from these infants to infants who are near-term. The literature on EEG and outcome includes all grades of prematurity. While it is very clear that EEG develops with gestational age as a proxy of ‘maturation’, it is not clear that any adverse effect should have a gestational age-specific signature. So, while it is important to adjust for gestational age in the statistical analysis, it may be fair to add evidence across studies of different gestational age bands. The EEG changes even faster in preterm infants after birth,^27,28^ possibly by the exposure to the abnormal extrauterine environment, but here we focus on ‘early’ EEG, i.e., recorded within the first week of life.

Several causal paths can be imagined to explain the association of the features of early EEG in preterm infants. First, EEG can be affected by genetic variation, or by preceding adversities such as intrauterine and/or perinatal complications that also affect the neurodevelopmental prospects. This was the rationale for the use of EEG at the end of the third day of life as one of the outcomes in the SafeBoosC-II trial to test the short-term effects of reducing the burden of cerebral hypo-or hyperoxia. Second, EEG can be affected during acute metabolic compromise, including oxygen deficiency, which may also have long-term consequences. It is likely, however, that the thresholds of cerebral oxygenation for inducing EEG changes are higher than the thresholds for inducing cellular injury^29^ although direct evidence of this in preterm infants has been difficult to provide.^30–32^

As a research tool, early EEG may be used for targeting infants of higher risk for randomised trials of neuroprotection and in cohort studies to improve the timing of neurological abnormality for the study of potential causal factors with preventive potential.^33^ The fact that it is single-channel, fully automated, and applicable to raw EEG data recorded by different devices may allow large-scale, multi-centre studies to address a priori defined questions.

As a clinical tool for prediction at the bedside, the present metrics appear less informative. First, the finding that burst sharpness has statistically significant predictive value over-and-above gestational age, sex, and early cerebral brain ultrasound, i.e., may offer added value in the clinical setting, is weakened by the fact that the associations of brain injury diagnosed by early cUS and NDI was not as expected. Possibly due to the small numbers, mild/moderate brain injury had surprisingly high predictive value and severe brain injury surprisingly had little value. Furthermore, even taking the result at face value and although the area under the ROC curve was respectable at 0.77, this only translates to a likelihood ratio for a positive test (EEG-burst sharpness above a given threshold) of approximately 2. This means that a ‘high’ burst sharpness can double the odds of a given infant for moderate-or-severe NDI. By Bayesian rules, if, say, the odds—as judged from the available information on gestational age, sex and brain ultrasound in a given infant—before the EEG results—were 0.4 for and 0.6 against (i.e., odds 0.66), the odds would increase to 1.33 by adding the EEG information (0.57 for/0.43 against). Or a ‘normal’ EEG sharpness could decrease the odds from 0.4 to 0.33 (0.25 for/0.75 against). Would this be clinically important? It is unlikely since there is currently no specific treatment to reduce this risk. Possibly, at high risks of NDI, if the value of life support in a very ill preterm infant with a severe brain US abnormality was questioned, an EEG may have a role in decisions regarding redirection of care.

In conclusion, this study confirms the association between an early computational EEG-burst feature and long-term neurodevelopmental outcomes. Importantly, this was by a priori defined analysis. Although the intention was to examine the predictive value of EEG over-and-above prediction based on gestational age and brain injury, the associations between brain injury and neurodevelopmental impairment were not as expected, so this result needs confirmation by new research.

## Data Availability

Anonymised patient data are available for research from the corresponding author. Consent was not obtained, but data will be anonymised and identifying characteristics removed from the dataset to minimise the risk of re-identification.
